# Efficient C–H bond activations *via* O_2_ cleavage by a dianionic cobalt(ii) complex†
†Electronic supplementary information (ESI) available: Experimental procedures, spectroscopic and electrochemical figures. CCDC 980872–980875. For ESI and crystallographic data in CIF or other electronic format see DOI: 10.1039/c4sc00108g



**DOI:** 10.1039/c4sc00108g

**Published:** 2014-05-20

**Authors:** Andy I. Nguyen, Ryan G. Hadt, Edward I. Solomon, T. Don Tilley

**Affiliations:** a Department of Chemistry , University of California at Berkeley , Berkeley , California 94720-1460 , USA . Email: tdtilley@berkeley.edu; b Department of Chemistry , Stanford University , Stanford , California 94305 , USA . Email: edward.solomon@stanford.edu

## Abstract

A dianionic, square planar cobalt(ii) complex reacts with O_2_ in the presence of acetonitrile to give a cyanomethylcobalt(iii) complex formed by C–H bond cleavage. Interestingly, PhIO and *p*-tolyl azide react similarly to give the same cyanomethylcobalt(iii) complex. Competition studies with various hydrocarbon substrates indicate that the rate of C–H bond cleavage greatly depends on the p*K*_a_ of the C–H bond, rather than on the C–H bond dissociation energy. Kinetic isotope experiments reveal a moderate KIE value of *ca.* 3.5 using either O_2_ or PhIO. The possible involvement of a cobalt(iv) oxo species in this chemistry is discussed.

## Abstract 2

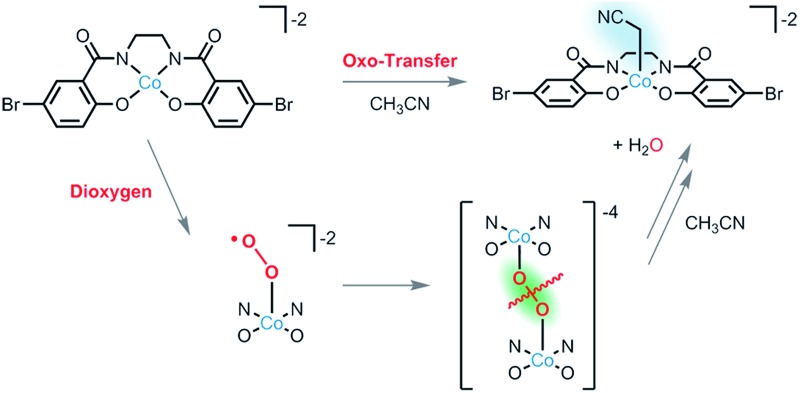
A new dianionic cobalt(ii) complex demonstrates parallel C–H bond activation reactivity with dioxygen and oxo-transfer reagents.

## Introduction

An important challenge for the development of new catalytic oxidations is the utilization of O_2_ from the air, as this reactant should enable the most atom-economical, cost effective and environmentally benign oxidations. In this context, the development of cobalt-based catalysts is particularly promising, since several types of homogeneous and heterogeneous cobalt catalysts are already known to utilize oxygen in oxidations of organic compounds.^1^ Much of this chemistry is thought to proceed *via* peroxy intermediates that initiate the generation of organic radicals.^2^ However, high-valent cobalt oxo intermediates have been postulated for certain oxidations, such as benzylic alcohol oxidation, amine oxidation, and epoxidation.^1^ Despite abundant circumstantial evidence for cobalt-mediated O_2_ cleavage, there has been no observation of a cobalt oxo species as the direct product of this type of O_2_ activation. Notably, the generation of a cobalt(iv) oxo intermediate *via* O_2_ cleavage would seem to be difficult given ligand field arguments,^3^ and the fact that numerous cobalt(ii) complexes have been shown to reversibly bind O_2_ to give superoxo or μ-peroxo complexes without O_2_ cleavage.^6^

**Scheme 1:**
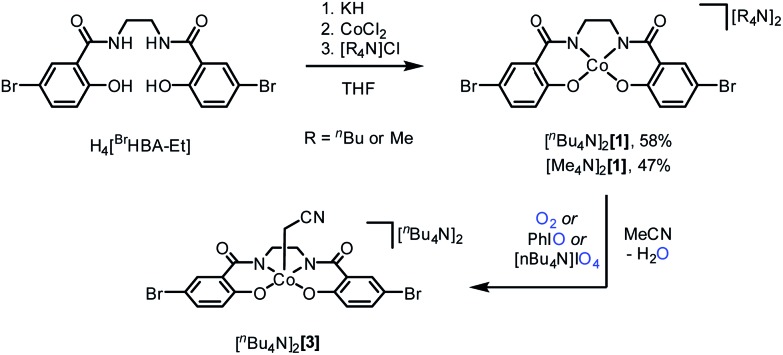
Synthesis of [^*n*^Bu_4_N]_2_ **1**, [^*n*^Me_4_N]_2_ **1**, and [^*n*^Bu_4_N]_2_ **3**.

This contribution describes attempts to promote O–O bond cleavage in a μ-peroxo intermediate to access a high-valent oxo species, with use of a tetraanionic ligand. The tetraanionic ligand ^Br^HBA-Et (Scheme 1) provides a strong ligand field that features hard donor atoms, and ligands of this type have been shown to support a number of metal complexes in high oxidation states.^7^ This approach has led to observation of clean C–H bond activations of acetonitrile and other hydrocarbons upon reaction of a dianionic cobalt(ii) complex with O_2_. Furthermore, the use of oxo- and imido-transfer agents is shown to produce reactive intermediates with similar characteristics, implying that putative cobalt(iv) oxo and imido complexes^5^ may be generated in these cases. Most importantly, this report describes a cobalt system that utilizes O_2_ cleavage in promoting unusual C–H bond activations.

## Results and discussion

**Fig. 1:**
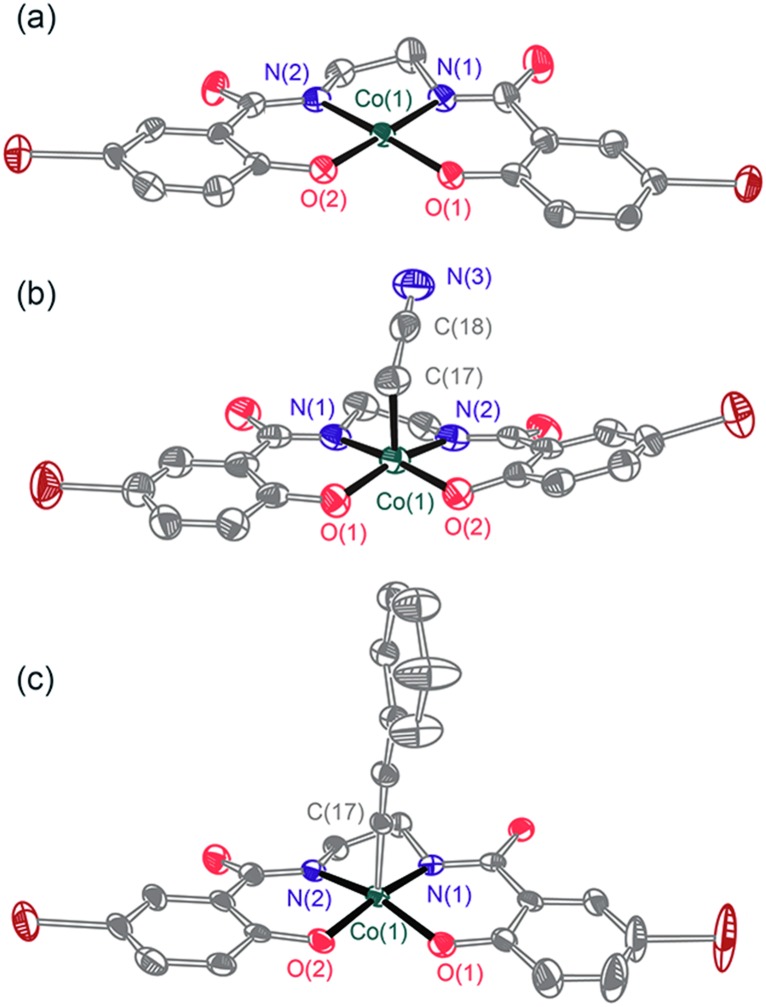
ORTEP diagrams of (a) [^*n*^Bu_4_N]_2_ **1**, (b) [^*n*^Bu_4_N]_2_ **3**, and (c) [^*n*^Bu_4_N]_2_ **4**. Thermal ellipsoids are drawn at 50%, and the solvent molecule, [^*n*^Bu_4_N]^+^ cations, and hydrogen atoms are omitted for clarity.

Deprotonation of (^Br^HBA-Et)H_4_, *N*,*N*′-(ethane-1,2-diyl)bis(5-bromo-2-hydroxybenzamide), with four equivalents of KH in THF, followed by treatment with CoCl_2_ and then salt metathesis with a tetraalkylammonium (^*n*^Bu_4_N^+^ or Me_4_N^+^) chloride gave the dianionic complexes [^*n*^Bu_4_N]_2_[(^Br^HBA-Et)Co] ([^*n*^Bu_4_N]_2_ **1**) and [Me_4_N]_2_[(^Br^HBA-Et)Co] ([Me_4_N]_2_ **1**) as orange, crystalline solids in 58% and 47% yields, respectively (Scheme 1). The complex [^*n*^Bu_4_N]_2_ **1** is a low spin, *S* = 1/2 Co(ii) complex, as demonstrated by the solution magnetic moment (1.98 *μ*_B_, 298 K). A rhombic signal in the 77 K X-band EPR spectrum (*g*_1_ = 1.956, *g*_2_ = 2.29, *g*_3_ = 3.15) with hyperfine coupling to the *I* = 7/2 ^59^Co nucleus (|*A*_1_| = 81 MHz, |*A*_2_| = 285 MHz, |*A*_3_| = 300 MHz) is consistent with an (*xy*)^2^(*z* ^2^)^2^(*yz*)^2^(*xz*)^1^ or (*xy*)^2^(*z* ^2^)^2^(*xz*)^2^(*yz*)^1^ ground state (ESI, Fig. S3†). Single crystal X-ray diffraction revealed that [^*n*^Bu_4_N]_2_ **1** contains a nearly square planar cobalt center (sum of angles about Co = 360.2(4)°), with Co–N and Co–O bond distances that both average to 1.87 Å (Fig. 1). Cyclic voltammetry reveals a reversible one-electron redox event at *E*_1/2_ = –0.22 V (*vs.* NHE) corresponding to the Co(ii)/Co(iii) couple (ESI, Fig. S6†). This redox couple is more negative than that for cobalt complexes supported by salen^8*a*^ (*E*_1/2_ = +0.25 V *vs.* NHE), porphyrin^8*b*^ (*E*_1/2_ = +0.68 V *vs.* NHE), corrole^8*c*^ (*E*_1/2_ = +0.03 V *vs.* NHE), 1,1′-(ethane-1,2-diyl)bis(3-*tert*-butylurea)^4*a*^ (*E*_1/2_ = –0.086 V *vs.* NHE), and 2,2′,2′-nitrilo-tris(*N*-isopropylacetamide)^4*b*^ (*E*_1/2_ = +0.44 V *vs.* NHE) ligands.

The one-electron oxidation of [^*n*^Bu_4_N]_2_ **1** by AgCl in THF afforded the Co(iii) complex [^*n*^Bu_4_N][(^Br^HBA-Et)Co] ([^*n*^Bu_4_N]**2**), which gives a deep purple solution in acetonitrile (*λ*_max_ = 560 nm, *ε* = 6800 M^–1^ cm^–1^; 98% isolated yield). Solution magnetic moment measurements characterize [^*n*^Bu_4_N]**2** as having an *S* = 1 (2.88 *μ*_B_, 298 K) ground state. X-ray crystallography (ESI, Fig. S1†) reveals that Co is in a nearly square planar geometry (sum of angles about Co = 360.5(2)°). The average Co–N and Co–O bond distances are contracted to 1.84 Å and 1.83 Å, respectively, relative to analogous values for [^*n*^Bu_4_N]_2_ **1**. Presumably, the electron-rich nature of the ligand in [**2**]^–^ stabilizes the four-coordinate cobalt(iii) center and substantially reduces its Lewis acidity. This geometry contrasts with that of most cobalt(iii) compounds which prefer an octahedral coordination environment with a diamagnetic ground state.

Reaction of 0.25 equivalents of dry dioxygen with [^*n*^Bu_4_N]_2_ **1** in acetonitrile-*d*_3_ resulted in a rapid color change from orange to dark brown, and generation of [^*n*^Bu_4_N]_2_ **3**-*d*_2_ in 88% yield by ^1^H NMR spectroscopy with respect to [^*n*^Bu_4_N]_2_ **1**. Use of excess dry dioxygen (1 atm) in the reaction with [^*n*^Bu_4_N]_2_ **1** in acetonitrile produced [^*n*^Bu_4_N]_2_ **3** in only 50% yield with respect to [^*n*^Bu_4_N]_2_ **1**. This lower yield is probably due to oxidation of the cyanomethyl ligand of [^*n*^Bu_4_N]_2_ **3** by the excess O_2_, to form [^*n*^Bu_4_N]**2** which was isolated from the reaction solution in 12% yield and identified by NMR spectroscopy. The complex [^*n*^Bu_4_N]_2_ **3**, isolated as green crystals, was shown by X-ray crystallography to possess a cyanomethyl ligand (Fig. 1b). Thus, reaction of [^*n*^Bu_4_N]_2_ **1** with O_2_ results in activation of the relatively strong C–H bond of acetonitrile (BDE_C–H_ = 96 kcal mol^–1^, p*K*_a_ = 31.1 in DMSO).

Interestingly, reactions of [^*n*^Bu_4_N]_2_ **1** with oxo-transfer reagents also resulted in formation of [^*n*^Bu_4_N]_2_ **3**. Thus, reaction of 0.5 of an equivalent of tetra-*n*-butylammonium periodate, [^*n*^Bu_4_N]IO_4_, with [^*n*^Bu_4_N]_2_ **1** in acetonitrile resulted in quantitative formation of [^*n*^Bu_4_N]_2_ **3** after 30 min (by ^1^H NMR spectroscopy). A control experiment using potassium iodate (KIO_3_) and 18-crown-6 with [^*n*^Bu_4_N]_2_ **1** in acetonitrile revealed no reaction over this time, demonstrating that all oxo-transfer reactivity is derived from the periodate anion. Similarly, reaction of 0.5 equivalents of PhIO with [^*n*^Bu_4_N]_2_ **1** gave [^*n*^Bu_4_N]_2_ **3** in 46% yield (by ^1^H NMR spectroscopy). The lower yield for this reaction is attributed to its heterogeneous nature.

Notably, Valentine and co-workers have shown that iodosylbenzene is activated as an oxo-transfer reagent in the presence of both redox-active and non-redox-active metal ions (*e.g.*, Co, and Zn), without the generation of an oxo ligand at the metal center. In these cases, iodosylbenzene undergoes Lewis-acid activation toward oxo-transfer to olefins (epoxidation) in the presence of metal salts in acetonitrile solvent, apparently with no activation of the latter.^9^ For comparison, a mixture of iodosylbenzene (1 equivalent), [^*n*^Bu_4_N]_2_ **1**, and cyclohexene in acetonitrile gave no transformation of the olefin during complete conversion of [^*n*^Bu_4_N]_2_ **1**, with formation of [^*n*^Bu_4_N]_2_ **3** in 46% yield, as monitored by ^1^H NMR spectroscopy and gas-chromatography-mass-spectrometry (GC-MS). Thus, any intermediate formed by the interaction of iodosylbenzene with [^*n*^Bu_4_N]_2_ **1** seems to have *low* electrophilicity and is unreactive toward cyclohexene. This appears to reflect the observed, low Lewis acidity for the cobalt(ii) center of [^*n*^Bu_4_N]_2_ **1**, with its strongly donating ligand and dianionic nature. Thus, Lewis acid activation of iodosylbenzene seems unlikely with [^*n*^Bu_4_N]_2_ **1**, and the highly reducing nature of this complex is more consistent with a redox event and oxo-transfer from iodosylbenzene. Thus, the observed reaction chemistry appears to suggest that a cobalt oxo intermediate forms upon reaction with O_2_ or oxo-transfer reagents.

To investigate the nature of the C–H bond activation step, a competition experiment between toluene (BDE_C–H_ = 86 kcal mol^–1^, p*K*_a_ = 41 in DMSO) and acetonitrile-*d*_3_ as substrates (1 : 1 molar mixture) was performed using PhIO or O_2_ as the oxidant. Significantly, [^*n*^Bu_4_N]_2_ **3**-*d*_2_ was the only organocobalt species observed by ^1^H NMR spectroscopy, and no trace of a toluene-activated product was detected. By ^2^H NMR spectroscopy, the reaction mixture contained no detectable amounts of deuterated toluene products that might form *via* initial toluene activation, followed by metathesis with acetonitrile-*d*_3_. In addition, a competition experiment involving 10 equivalents of 9,10-dihydroanthracene, a more acidic substrate (BDE_C–H_ = 78 kcal mol^–1^, p*K*_a_ = 30 in DMSO),^10^ in acetonitrile-*d*_3_ solvent produced anthracene (13%) and 9,10-anthraquinone (12%) using PhIO or O_2_, respectively, as oxidant.^11^ The formation of 9,10-anthraquinone with O_2_ as the oxidant may result from reaction of ^3^O_2_ with the 9-anthracenyl radical formed by initial C–H bond abstraction.^12^ Thus, C–H bond activations in this system appear to be promoted by acidic character in the C–H bond. This was further indicated by reactions of [^*n*^Bu_4_N]_2_ **1** in acetonitrile with O_2_ or PhIO in the presence of phenylacetylene (10 equivalents), which contains a very acidic but strong C–H bond (BDE_C–H_ = ∼133 kcal mol^–1^, p*K*_a_ = 28 in DMSO).^13^ This reaction quantitatively (by ^1^H NMR spectroscopy) forms the diamagnetic, green cobalt alkynyl complex [^*n*^Bu_4_N]_2_[(^Br^HBA-Et)Co–C≡CPh] ([^*n*^Bu_4_N]_2_ **4**, *ν*_C≡C_ = 2106 cm^–1^). The structure of [^*n*^Bu_4_N]_2_ **4** was confirmed by X-ray crystallography (Fig. 1c). The strong preference for more acidic substrates suggests that in the C–H bond activation step, proton transfer may precede electron transfer (*vide infra*).

A kinetic isotope effect (KIE) for reaction of acetonitrile in the presence of [^*n*^Bu_4_N]_2_ **1** and O_2_ was obtained from a competition experiment involving a 1 : 1 mixture of acetonitrile and acetonitrile-*d*_3_. Quantification of the [^*n*^Bu_4_N]_2_ **3**/[^*n*^Bu_4_N]_2_ **3-** *d*_2_ ratio by high-resolution electrospray mass spectrometry gave a KIE value of 3.3(2). Interestingly, the analogous reaction with PhIO as the oxidant provided a KIE value that is essentially identical, 3.6(2). This moderate primary KIE value is consistent with an early or late transition state for the C–H bond activation step, and heterolytic C–H bond cleavage. Coupled with the selectivities described above, these results suggest that a common intermediate is formed by reactions of PhIO or O_2_ with [^*n*^Bu_4_N]_2_ **1**, which activates C–H bonds by a heterolytic mechanism involving considerable proton-transfer character. Note that highly basic metal oxo complexes are expected to exhibit a moderate primary KIE in C–H bond activations.^14^

**Fig. 2:**
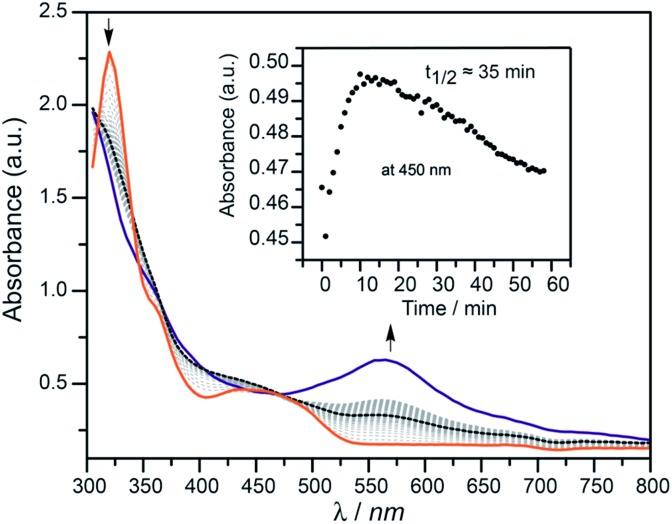
UV-vis time trace of [^*n*^Bu_4_N]_2_ **1** reaction with 1 atm O_2_ at –63 °C in 1 : 1 MeCN–THF. Orange trace is [^*n*^Bu_4_N]_2_ **1**; purple trace is at *t* = 60 min; dashed-black trace is *t* = 10 min.

**Scheme 2:**
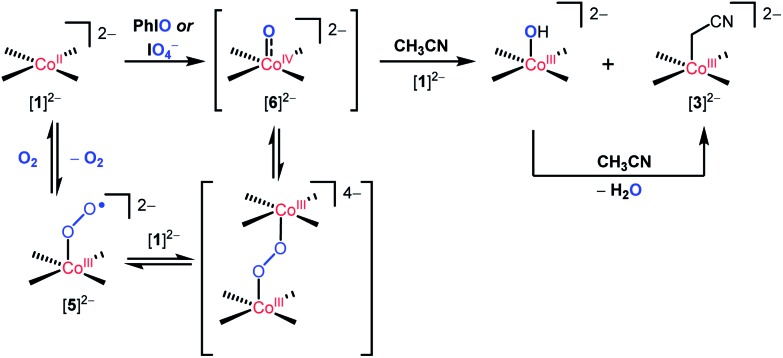
Proposed mechanism for the formation of [**3**]^2–^ by the reaction of [**1**]^2–^ with O_2_ or oxo-transfer reagents.

In an attempt to observe an intermediate in the O_2_-mediated activation of acetonitrile, a 1 : 1 THF–MeCN solution of [^*n*^Bu_4_N]_2_ **1** at –63 °C was treated with O_2_ (1 atm) and the reaction progress was followed by UV-vis spectroscopy. After 10 min, a new absorbance at *ca.* 400–450 nm, which decayed over the course of 1 h (*t*_1/2_ ≈ 35 min), was observed (Fig. 2). EPR spectroscopy (X-band, 77 K; ESI, Fig. S4†) was used to characterize this intermediate, generated by addition of dry O_2_ at room temperature to an *n*-butyronitrile solution of [^*n*^Bu_4_N]_2_ **1** in the EPR tube. This experiment allowed observation of a new *S* = 1/2 signal with hyperfine coupling to ^59^Co (*g*_1_ = 2.026, *g*_2_ = 2.028, *g*_3_ = 2.13, |*A*_1_| = 57 MHz, |*A*_2_| = 43 MHz, |*A*_3_| = 85 MHz), the appearance of which after 30 seconds was accompanied by elimination of the signal for [^*n*^Bu_4_N]_2_ **1**. After 10 minutes at room temperature, the intensity of the new signal diminished (to ∼10% of the original intensity, by integration of the EPR signal) with no appearance of new signals, consistent with the formation of EPR-silent products. However, when the solution was degassed by a freeze–pump–thaw cycle after acquisition of the first spectrum, the *S* = 1/2 signal diminished and the signal for [^*n*^Bu_4_N]_2_ **1** increased (ESI, Fig. S5†). This behavior is consistent with reversible O_2_ coordination to the cobalt(ii) center to form the η^1^-superoxo complex [^*n*^Bu_4_N]_2_[(^Br^HBA-Et)Co(O_2_)], [^*n*^Bu_4_N]_2_ **5** (Scheme 2).^4^ In addition, analysis of the reaction mixture of [Me_4_N]_2_ **1** with O_2_ in acetonitrile after approximately 10 seconds at room temperature by high-resolution ESI-MS revealed the presence of a dianion with *m*/*z* = 264.42, and an isotopic distribution consistent with the terminal cobalt(iv) oxo complex [^*n*^Bu_4_N]_2_[(^Br^HBA-Et)CoO], [^*n*^Bu_4_N]_2_ **6** (ESI, Fig. S4†).

The observations of [^*n*^Bu_4_N]_2_ **5** and [^*n*^Bu_4_N]_2_ **6** implicate these species as potential intermediates that directly engage in C–H bond-cleavage reactions. However, the results described above are most consistent with involvement of the oxo complex [**6**]^2–^ in this chemistry. Firstly, metal superoxo complexes often exhibit much higher *k*_H_/*k*_D_ values in intermolecular C–H bond activations (a range of *k*_H_/*k*_D_ = 6.3 to 50 is typical).^15^ The substrate competition experiments are consistent with initial proton abstraction from a C–H bond, but the p*K*_a_ of hydrogen superoxide (p*K*_a_ = 12 in DMF)^16,17^ suggests that such species should not be basic enough to accomplish such deprotonations. Furthermore, the observed reaction chemistry for O_2_ closely parallels that observed with oxo-transfer reagents PhIO and IO_4_^–^, suggesting a cobalt(iv) oxo species as a common intermediate. Thus, the reaction with O_2_ presumably involves binding of cobalt(ii) to the initially formed superoxo complex [**5**]^2–^, followed by O–O bond cleavage to generate the oxo complex [**6**]^2–^. This oxo complex is presumed to react with hydrocarbons *via* proton- and electron-transfers to produce hydroxide and the cobalt(iii) complex [**2**]^2–^. This would generate the cyanomethyl radical NCCH_2_˙, and rapid trapping of this species by [**1**]^2–^ would give [**3**]^2–^. The cobalt(iii) hydroxide species, proposed as the direct product of a proton-coupled-electron-transfer to [**6**]^2–^, is expected to provide a second pathway to the [**3**]^2–^ product, *via* deprotonation of acetonitrile. This hypothesis is supported by the observed reaction of [**2**]^2–^ with (18-crown-6⊂K)OH in acetonitrile over 1 h, to give [**3**]^2–^ in quantitative yield.

**Scheme 3:**
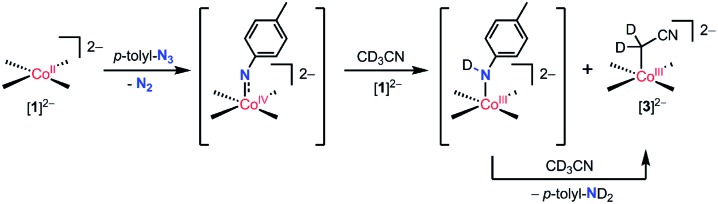
Proposed mechanism for the formation of [**3**]^2–^ by the reaction of [**1**]^2–^ with tolyl azide.

The reaction of [^*n*^Bu_4_N]_2_ **1** with the oxo-transfer reagents PhIO and IO_4_^–^ suggest the possibility of a putative cobalt(iv) oxo complex. Consistent with this hypothesis, [^*n*^Bu_4_N]_2_ **1** was observed to react with *p*-tolyl azide, a nitrene transfer reagent, in acetonitrile-*d*_3_ to cleanly produce [^*n*^Bu_4_N]_2_ **3** and 1 equiv of toluidine-*d*_2_ (by ^1^H NMR and ^2^H NMR spectroscopy). This reaction is tentatively proposed to proceed *via* a cobalt(iv) imido species and a mechanism analogous to that proposed above for the cobalt(iv) oxo species (Scheme 3).

## Conclusions

In summary, the reaction of O_2_ with [^*n*^Bu_4_N]_2_ **1** forms a cyanomethylcobalt(iii) complex, [^*n*^Bu_4_N]_2_ **3**, that results from an intermolecular C–H bond activation of acetonitrile. Oxo- and nitrene-transfer reagents are observed to induce the same reactivity, suggesting that cobalt(iv)-oxo and -imido species are key intermediates. Note that Schaefer and coworkers reported that, in the presence of O_2_, a (salen)cobalt(ii) species activates acetone to form a Co–C bond. No mechanistic details were provided, but this process may proceed *via* a cobalt-oxo intermediate that abstracts proton from acetone (which is about 7 orders of magnitude more acidic than acetonitrile).^18^ The well-behaved system described above is expected to provide opportunities to establish mechanistic details for O_2_ activation by cobalt(ii), and perhaps the microscopic reverse, O–O bond formation. Along these lines, future efforts will target kinetic and low-temperature spectroscopic studies to better characterize the intermediates resulting from O_2_ activation.
