# Supplementary material for: Efficient C–H bond activations via O2 cleavage by a dianionic cobalt(ii) complex
Source: Chem Sci. 2014 May 20;5(7):2874–8. doi: 10.1039/c4sc00108g (PMC4111274; doi:10.1039/c4sc00108g)
Supplement: Supplementary file 1 [file SC-005-C4SC00108G-s001.pdf]

Electronic Supplementary Material (ESI) for Chemical Science.
This journal is © The Royal Society of Chemistry 2014

Supporting Information

Efficient C-H Bond Activations *via* O_2 Cleavage by a Dianionic Cobalt(II) Complex

*Andy I. Nguyen[1], Ryan G. Hadt[2], Edward I. Solomon[2], T. Don Tilley[1]**

[1]Department of Chemistry, University of California at Berkeley, Berkeley, California 94720-1460.

[2]Department of Chemistry, Stanford University, Stanford, California 94305

*Email: tdtilley@berkeley.edu

1. Experimental Details

General Considerations

All manipulations were carried out using Schlenk techniques[1] under a purified N_2 atmosphere or in a Vacuum Atmospheres drybox. Solvents (spectroscopic grade) were purchased from Aldrich. Dry THF was prepared by passage through a VAC drying column. Acetonitrile was dried with CaH_2, and then fractionally distilled from CaH_2 under N_2. Acetonitrile used for crystallization was vacuum transferred from P_2O_5. *n*-Butyronitrile (^{n}PrCN) was dried by distillation over CaH_2. Dry solvents were stored under N_2 in Straus flasks. Cobalt(II) chloride, potassium hydride, 5-bromo-salicylic acid, ethylenediamine, iodosylbenzene (PhIO), 9,10-dihydroanthracene, and triphenylphosphine were purchased from Aldrich and used as received. NMR spectra were recorded on Bruker AVB-400, AVQ-400 and AV-300 spectrometers at room temperature. d_3-MeCN and d_6-DMSO were purchased from Cambridge Isotopes and dried over 3Å sieves under N_2. ^{1}H NMR spectra were referenced to residual protio solvent peaks (δ 2.94 for CD_3CN, δ 2.50 for $(CD_3)_2SO$). $^{13}C\{^1H\}$ NMR spectra were referenced to solvent resonances (δ

118.26 for CD_3CN, δ 39.51 for $(CD_3)_2SO$). Elemental analyses were carried out by the College of Chemistry Microanalytical Laboratory at the University of California, Berkeley. Infrared spectra were recorded on a Bruker Alpha P attenuated total reflectance instrument. UV-vis spectra were obtained on a Varian Cary 300 Bio or Varian Cary 50 BIO UV−visible spectrophotometer with a Unisoko cryostat attachment for stirring and temperature control in 1-cm air-free quartz cells. X-band electron paramagnetic resonance (EPR) spectra were obtained using a Bruker EMX spectrometer. X-band spectra were obtained at 77 K in a liquid nitrogen finger dewar using an ER 041 XG microwave bridge and an ER 4102ST cavity (parameters for recording the X-band EPR data: 9.63 GHz frequency, 1 mW microwave power, and 10 G modulation amplitude). X-band data were simulated using the Easy Spin program. Mass spectrometry measurements were performed at the QB3 Mass Spectrometry Facility at UC Berkeley using a quadrupole time-of-flight mass spectrometer (Q-TOF Premier, Waters, Milford, MA) that was equipped with an electrospray ionization (ESI) source. ESI source voltages were adjusted for optimum desolvation and transmission of ions of interest prior to recording mass spectra. Cyclic voltammograms were obtained with a BASi Epsilon potentiostat at room temperature using a glassy carbon working electrode (7 mm diameter), a platinum counter electrode, and a silver wire floating reference. All potentials were referenced against an $[Cp_2Fe]^{0/+}$ internal standard in acetonitrile.

Preparation of (^{Br}HBA-Et)H_4, *N*,*N'*-(ethane-1,2-diyl)*bis*(5-bromo-2-hydroxybenzamide). 5-Bromo-salicylic acid (5.4 g, 25 mmol) was combined with acetic anhydride (5 mL) and sulfuric acid (3 drops). The mixture was heated to 80 °C for 10 min. Water (100 mL) was added to the solid mass and the resulting solid was collected by filtration (5.6 g). The solid was dissolved in oxalyl chloride (6 mL) and DMF (2 drops) over 1 h. The volatile materials were removed *in*

vacuo and the resulting residue was triturated twice with dichloromethane to give a white solid. The solid was dissolved in 100 mL of THF and the resulting solution was cooled to 0 °C. Ethylenediamine (0.72 mL, 11 mmol) was added dropwise and the resulting solution was stirred for 30 min. Triethylamine (4.5 mL, 32 mmol) in 100 mL of THF was added and the stirring was continued for 45 min at room temperature. The solution was filtered and the filtrate was evaporated to dryness and then washed with cold ethanol (0°C, 50 mL) to give a white solid. The solid from the last filtration was washed with 500 mL of water. The combined solids were dissolved in a solvent mixture of 200 mL water / 150 mL ethanol containing 1.5 g (37 mmol) of sodium hydroxide. The solution was refluxed for 30 min and then quenched with 3.5 mL of HCl (12.1 M). The resulting mixture was cooled to 0 °C and then filtered to give the product as a white solid (3.12 g, 55%).

^{1}H NMR (400 MHz, d_6-DMSO) δ: 3.49 (m, 4H, –CH_2CH_2–), 6.89 (d, 2H, $^3J_{HH}$ = 8.7, aryl-*H*), 7.55 (dd, 2H, $^3J_{HH}$ = 8.7, $^4J_{HH}$ = 2.2, aryl-*H*), 8.03 (d, 2H, $^4J_{HH}$ = 2.2, aryl-*H*), 9.01 (t, 2H, $^3J_{HH}$ = 5.3, N–*H*), 12.53 (s, 2H, O–*H*); HRMS (ESI) for $C_{16}H_{13}Br_2N_2O_4$ $[M - H^+]^-$ 456.92, found 456.9214.

Preparation of $[^n\mathbf{Bu_4N}]_2[(^{Br}\mathbf{HBA\text{-}Et})\mathbf{Co}]$ ($[^n$Bu$_4$N$]_2$1). Potassium hydride (0.102 g, 2.54 mmol), **$(^{Br}$HBA-Et$)$H$_4$** (0.292 g, 0.640), and 10 mL of THF were combined in a 20 mL vial, and the resulting reaction mixture was stirred for 16 h. $CoCl_2$ (0.083 g, 0.640 mmol) was then added and the dark green solution was stirred for 16 h. Tetra-*n*-butylammonium chloride (0.354 g, 1.27 mmol) was then added and the stirring was continued for 2 h. The orange precipitate was collected by filtration and extracted into 15 mL of acetonitrile. The extract was evaporated *in vacuo* to give an orange crystalline solid (0.371 g, 58%). Anal. Calcd for $C_{48}H_{82}Br_2CoN_4O_4$: C, 57.77; H, 8.28; N, 5.61. Found: C, 57.17; H, 8.30; N, 5.28. IR (neat) ν/cm^{-1}: 2959, 2931, 2873,

1577, 1550, 1489, 1460, 1406, 1520, 1326, 1263, 1228, 1143, 1110, 882, 819, 681, 736, 656, 625, 535, 465, 419. UV-vis (MeCN) λ_{max}/nm (ε/M^{-1} cm^{-1}): 450 (2200), 320 (17,000), 217 (62,000). Magnetic Susceptibility (298 K, MeCN) μ_B: 1.98.

Preparation of [^{n}Me$_4$N]$_2$[(^{Br}HBA-Et)Co] ([Me$_4$N]$_2$**1**•2CH$_3$CN**).** Potassium hydride (0.145 g, 3.61 mmol), **(^{Br}HBA-Et)H$_4$** (0.4127 g, 0.900 mmol), and 10 mL of THF were combined in a 20 mL vial, and the resulting reaction mixture was stirred for 16 h. $CoCl_2$ (0.117 g, 0.900 mmol) was then added and the resulting green solution was stirred for 3 h. Tetra-*n*-methylammonium chloride (0.197 g, 1.80 mmol) was then added and stirring was continued for 16 h. The orange precipitate was collected by filtration and extracted into 15 mL of MeCN. The extract was evaporated *in vacuo* to give an orange crystalline solid (0.293 g, 44%). Anal. Calcd for $C_{48}H_{82}Br_2CoN_4O_4$•2CH$_3$CN: C, 45.24; H, 5.42; N, 11.30. Found: C, 45.0; H, 5.51; N, 11.0.

Preparation of [^{n}Bu$_4$N][(^{Br}HBA-Et)Co] ([^{n}Bu$_4$N]**2).** AgCl (0.0291 g, 0.205 mmol) was added to a slurry of [^{n}Bu$_4$N]$_2$**1** (0.205 g, 0.205 mmol) in 10 mL of THF. The reaction mixture was stirred for 45 minutes, and then 3 mL of Et_2O was added to precipitate [^{n}Bu$_4$N]Cl. Filtration of the reaction mixture through a celite plug and evaporation of the resulting purple solution afforded a purple solid (0.155 g, 98%). Figure S1 shows the x-ray crystal structure with selected bond lengths.

Anal. Calcd for $C_{32}H_{46}Br_2CoN_3O_4$: C, 50.87; H, 6.14; N, 5.56. Found: C, 50.0; H, 5.90; N, 5.29. ^{1}H NMR (400 MHz, CD$_3$CN) δ: -28.05 (s, 2H, aryl-*H*), 0.72 (t, 12H, $^3J_{HH}$ = 6.5, (C*H*$_3$CH$_2$CH$_2$CH$_2$)$_4$N), 1.05 (sex, 8H, $^3J_{HH}$ = 6.5, (CH$_3$C*H*$_2$CH$_2$CH$_2$)$_4$N), 1.28 (br, 8H, (CH$_3$CH$_2$C*H*$_2$CH$_2$)$_4$N), 2.73 (t, 8H, $^3J_{HH}$ = 6.3, (C*H*$_3$CH$_2$CH$_2$C*H*$_2$)$_4$N), 19.00 (s, 2H, aryl-*H*), 29.93 (s, 2H, aryl-*H*), 318.04 (s, 2H, –C*H*$_2$C*H*$_2$–). IR (neat) ν/cm^{-1}: 2956, 2931, 2868, 1580, 1552, 1520, 1456, 1409, 1298, 1145, 1093, 882, 821, 740, 685, 630, 583, 538, 487, 464, 424.

UV-vis (MeCN) λ_{max}/nm (ε/M^{-1} cm^{-1}): 560 (6800), 378 (3000), 303 (10,000), 250 (15,000), 214 (38,000). Magnetic Susceptibility (298 K, MeCN) μ_B: 2.88.

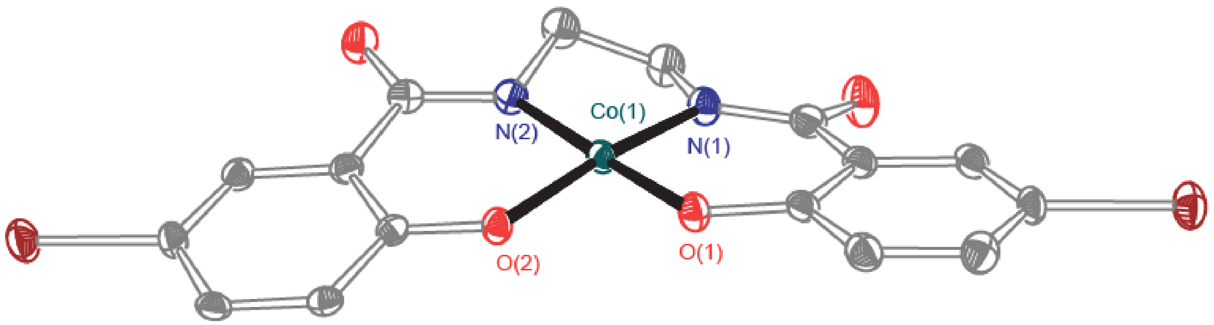

Figure S1. ORTEP diagram of [^{n}Bu$_4$N]$_2$**2**. Thermal ellipsoids are drawn at 50%, hydrogen atoms and ^{n}Bu$_4$N$^+$ cations are omitted for clarity. Selected bond distances (Å) and angles (deg) for [1]: N(1)–Co(1) 1.834(2), N(2)–Co(1) 1.836(2), O(1)–Co(1) 1.831(2), O(2)–Co(1) 1.830(2); O(2)–Co(1)–N(2) 94.90(9), N(2)–Co(1)–N(1) 86.41(10), N(1)–Co(1)–O(1) 95.22(9), O(2)–Co(1)–O(1) 83.98(8).

Preparation of [^{n}Bu$_4$N]$_2$[(^{Br}HBA-Et)Co(CH$_2$CN)] ([^{n}Bu$_4$N]$_2$3). A MeCN solution (5 mL) of [^{n}Bu$_4$N]$_2$**1** (0.107 g, 0.107 mmol) in a Schlenk tube was degassed with one freeze-pump-thaw cycle, and this solution was exposed to 1 atm of dry O_2 at room temperature. The reaction mixture was stirred for 2 h at room temperature to produce a dark brown solution. The volatile materials were removed *in vacuo* to afford a brown residue. The residue was dissolved in 3 mL of THF, and 3 mL of Et$_2$O was added to yield a green oily residue and a purple solution. The purple solution was decanted away from the oil, and evaporated *in vacuo* to give [^{n}Bu$_4$N]**[2]** (0.010 g, 12% based on [^{n}Bu$_4$N]$_2$**1**), identified by ^{1}H NMR spectroscopy. The green oil was stirred vigorously in 10 mL of Et$_2$O to give [^{n}Bu$_4$N]$_2$**3** as a green solid, which was collected by filtration (0.056 g, 50% based on [^{n}Bu$_4$N]$_2$**1**). Single crystals suitable for X-ray diffraction were obtained by vapor diffusion of Et$_2$O into a saturated solution of [^{n}Bu$_4$N]$_2$**3** in THF.

Anal. Calcd for $C_{50}H_{84}Br_2CoN_5O_4$: C, 57.86; H, 8.16; N, 6.75. Found: C, 57.58; H, 8.05; N, 7.07. ^{1}H NMR (400 MHz, CD_3CN) δ: 0.93 (t, 24H, $^3J_{HH}$ = 6.8, ($CH_3CH_2CH_2CH_2)_4N$), 1.31 (sex, 16H, $^3J_{HH}$ = 6.8, ($CH_3CH_2CH_2CH_2)_4N$), 1.53 (br, 16H, ($CH_3CH_2CH_2CH_2)_4N$), 1.54 (s, 2H, CH_2CN) 3.04 (br, 16H, ($CH_3CH_2CH_2CH_2)_4N$), 3.50 (m, 4H, –CH_2CH_2–), 6.93 (d, 2H, $^3J_{HH}$ = 8.4, aryl-*H*), 6.98 (dd, 2H, $^3J_{HH}$ = 8.4, $^4J_{HH}$ = 2, aryl-*H*), 8.01 (d, 2H, $^4J_{HH}$ = 2, aryl-*H*). $^{13}C\{^1H\}$ NMR (100.62 MHz, CD_3CN) δ: 13.72 (($CH_3CH_2CH_2CH_2)_4N$), 20.30 (($CH_3CH_2CH_2CH_2)_4N$), 24.20 (($CH_3CH_2CH_2CH_2)_4N$), 50.34 (–CH_2CH_2–), 59.15 (($CH_3CH_2CH_2CH_2)_4N$), 104.15 (aryl-*C*), 126.33 (aryl-*C*), 131.89 (aryl-*C*), 133.26 (aryl-*C*), 169.59 (aryl-*C*), 170.96 (*C*=O). IR (neat) ν/cm^{-1}: 2961, 2931, 2873, 2203 (C≡N), 1580, 1552, 1520, 1452, 1409, 1309, 1261, 1228, 1145, 1108, 1093, 1025, 882, 819, 739, 680, 648, 628, 535, 465, 440, 423. UV-vis (MeCN) λ_{max}/nm (ε/M^{-1} cm^{-1}): 800 (1300), 217 (68,000).

Generation of [^{n}Bu$_4$N]$_2$[(^{Br}HBA-Et)Co(CH$_2$CN)] ([^{n}Bu$_4$N]$_2$3) by stoichiometric O$_2$. In a J. Young tube, [^{n}Bu$_4$N]$_2$**1** (0.031 g, 0.031 mmol) was dissolved in 0.6 mL of CD_3CN. The solution was degassed by a freeze-pump-thaw cycle. A rubber septum with a needle was fitted on the J. Young tube and the headspace was degassed by pulling vacuum. A syringe containing 0.2 mL dry O_2 (0.0078 mmol) was inserted into the septum, and the J. Young seal was opened to allow the O_2 to diffuse into the tube. The reaction was complete after 16 h, and [^{n}Bu$_4$N]$_2$**3** was formed along 88% yield by ^{1}H NMR spectroscopy (with respect to [^{n}Bu$_4$N]$_2$**1**), using 1,4-dimethoxybenzene or [^{n}Bu$_4$N]$^+$ as an internal standard.

Generation of [^{n}Bu$_4$N]$_2$[(^{Br}HBA-Et)Co(CH$_2$CN)] ([^{n}Bu$_4$N]$_2$3) by PhIO. In a J. Young tube, [^{n}Bu$_4$N]$_2$**1** (0.037 g, 0.037 mmol) and PhIO (0.0041 g, 0.018 mmol) were dissolved in 0.6 mL of CD_3CN. The reaction was complete after 16 h, and [^{n}Bu$_4$N]$_2$**3** was formed along with

iodobenzene in quantitative yield by ^{1}H NMR spectroscopy (with respect to both iodosylbenzene and [^{n}Bu$_4$N]$_2$**1**), using 1,4-dimethoxybenzene or [^{n}Bu$_4$N]$^+$ as an internal standard.

Generation of [^{n}Bu$_4$N]$_2$[(^{Br}HBA-Et)Co(CH$_2$CN)] ([^{n}Bu$_4$N]$_2$3) by [^{n}Bu$_4$N]IO$_4$. In a J. Young tube, [^{n}Bu$_4$N]$_2$**1** (0.050 g, 0.050 mmol) was dissolved in ~1 mL of CD_3CN, and [^{n}Bu$_4$N]IO$_4$ (0.010 g, 0.025 mmol) was added slowly in small batches with stirring. The reaction was complete after 0.5 h, and [^{n}Bu$_4$N]$_2$**3** was formed in near quantitative yield (96% with respect to [^{n}Bu$_4$N]$_2$**1**) by ^{1}H NMR spectroscopy, using either 1,4-dimethoxybenzene (0.0055g) or [^{n}Bu$_4$N]$^+$ as an internal standard. A spectrum of this reaction is shown below as a representative of all ^{1}H NMR spectroscopy reactions in this paper:

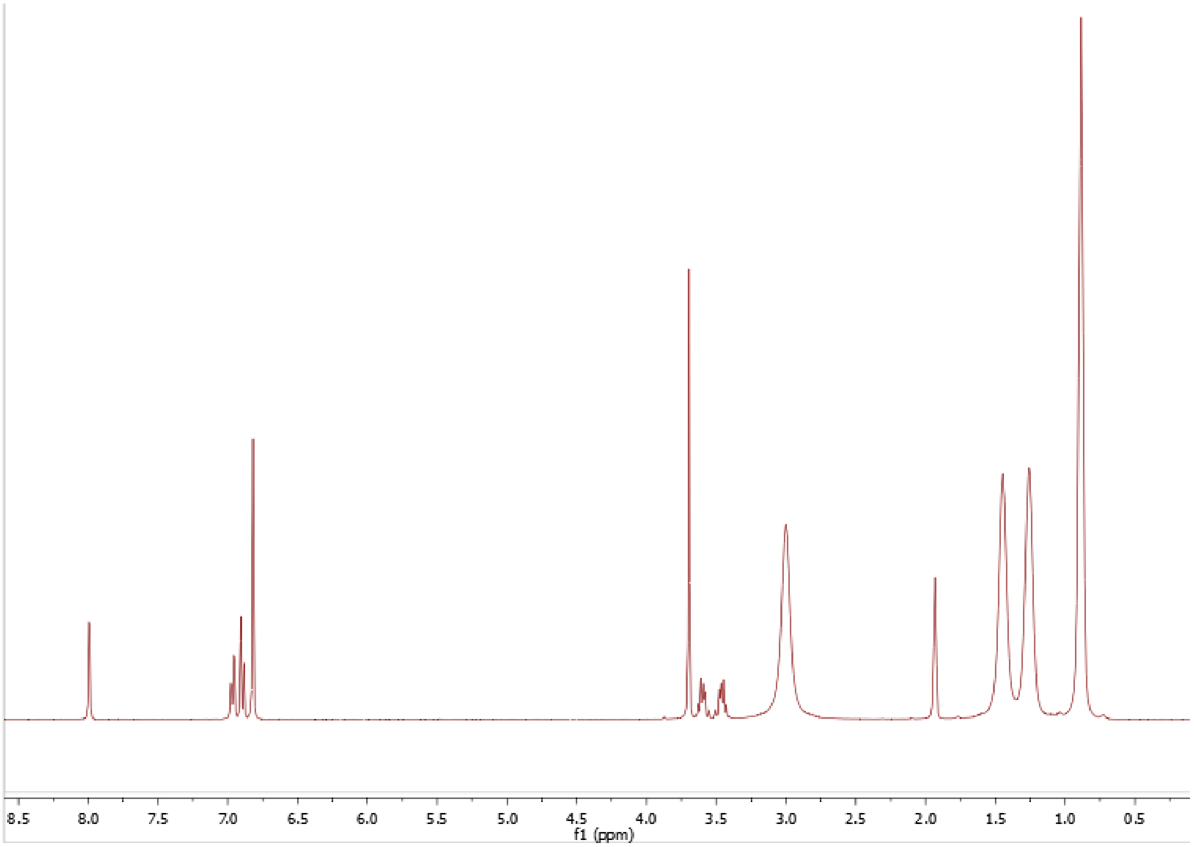

Preparation of [^{n}Bu$_4$N]$_2$[(^{Br}HBA-Et)Co(CCPh)] ([^{n}Bu$_4$N]$_2$4). To a MeCN solution (5 mL) of [^{n}Bu$_4$N]$_2$**1** (0.100 g, 0.107 mmol) was added phenylacetylene (0.102 g, 1.10 mmol). Iodosylbenzene (0.011 g, 0.050 mmol) was added, and the resulting reaction mixture was stirred

for 3 h. Diethyl ether (15 mL) was added to the solution and a green crystalline solid formed overnight. The mother liquor was decanted and the solid was dried in vacuo to yield 0.084 g (76%) of the product.

^{1}H NMR (400 MHz, CD_3CN) δ: 0.92 (br, 24H, $(CH_3CH_2CH_2CH_2)_4N$), 1.29 (br, 16H, $(CH_3CH_2CH_2CH_2)_4N$), 1.52 (br, 16H, $(CH_3CH_2CH_2CH_2)_4N$), 3.03 (br, 16H, $(CH_3CH_2CH_2CH_2)_4N$), 3.45 (m, 2H, $-CH_2CH_2-$), 3.67 (m, 2H, $-CH_2CH_2-$), 6.84–7.00 (m, 9H, aryl-*H*), 8.04 (br, 2H, aryl-*H*). $^{13}C\{^{1}H\}$ NMR (100.62 MHz, CD_3CN) δ: 13.85 ($(CH_3CH_2CH_2CH_2)_4N$), 20.29 ($(CH_3CH_2CH_2CH_2)_4N$), 24.29 ($(CH_3CH_2CH_2CH_2)_4N$), 51.15 ($-CH_2CH_2-$), 59.14 ($(CH_3CH_2CH_2CH_2)_4N$), 105.28 (aryl-*C*), 114.93 (Ph*C*≡C–), 125.51 (aryl-*C*), 125.83 (aryl-*C*), 127.26 (aryl-*C*), 128.83 (aryl-*C*), 132.24 (aryl-*C*), 132.93 (aryl-*C*), 133.04 (aryl-*C*), 168.94 (aryl-*C*), 171.41 (*C*=O). IR (neat) ν/cm^{-1}: 2958, 2934, 2870, 2106 (C≡C), 1576, 1555, 1519, 1458, 1440, 1410, 1317, 1265, 1229, 878, 814; HRMS (ESI) for $C_{24}H_{15}Br_2CoN_2O_4$ $[M]^{2-}$ m/z = 306.94, found 306.94.

Generation of $[^{n}Bu_4N]_2[(^{Br}HBA\text{-}Et)Co(CCPh)]$ ($[^{n}Bu_4N]_2$4) by O_2. A J. Young tube containing $[^{n}Bu_4N]_2$**1** (0.025 g, 0.025 mmol) and phenylacetylene (0.026 g, 0.25 mmol) was degassed by one freeze-pump-thaw cycle. Excess dry O_2 (1 atm) was added to the reaction mixture. After 1 h, a green solution was obtained. ^{1}H NMR spectroscopy showed quantitative formation of $[^{n}Bu_4N]_2$**4** by integration against 1,4-dimethoxybenzene internal standard (0.003 g, 0.022 mmol).

Reaction of $[^{n}Bu_4N]_2$1, KIO_3, and 18-crown-6 in MeCN. Potassium iodate (0.005 g, 0.025 mmol) was added to a MeCN solution (0.6 mL) containing $[^{n}Bu_4N]_2$**1** (0.050 g, 0.050 mmol) and 18-crown-6 (0.006 g, 0.025 mmol). The reaction was allowed to react for 16 h. No color change was observed indicating no generation of $[^{n}Bu_4N]_2$**3**.

Reaction of [^{n}Bu$_4$N]$_2$1, PhIO, and cyclohexene. PhIO (0.011 g, 0.050 mmol) was added to a d_3-MeCN solution (0.6 mL) containing [^{n}Bu$_4$N]$_2$**1** (0.050 g, 0.050 mmol) and excess cyclohexene (0.042 g, 0.50 mmol). The reaction was allowed to react for 16 h, and analyzed by ^{1}H NMR spectroscopy which showed a 46% yield of [^{n}Bu$_4$N]$_2$**3**, and no change in cyclohexene integration (no new shifts corresponding to any new organic products were observed either) using [^{n}Bu$_4$N]$^+$ as an internal standard. Gas-chromatography mass spectrometry only showed peaks for cyclohexene, 1,4-dimethoxybenzene, and iodobenzene.

Reaction of [^{n}Bu$_4$N]$_2$1, O$_2$, and 9,10-dihydroanthracene. Excess O_2 was added to a d_3-MeCN slurry (0.6 mL) containing [^{n}Bu$_4$N]$_2$**1** (0.0403 g, 0.0404 mmol) and 9,10-dihydroanthracene (0.072 g, 0.404 mmol). The reaction mixture was analyzed by ^{1}H NMR spectroscopy after 24 h, which showed a 12% yield of anthraquinone using [^{n}Bu$_4$N]$^+$ as an internal standard.

Reaction of [^{n}Bu$_4$N]$_2$1, PhIO, and 9,10-dihydroanthracene. PhIO (0.004 g, 0.020 mmol) was added to a d_3-MeCN slurry (0.6 mL) containing [^{n}Bu$_4$N]$_2$**1** (0.020 g, 0.020 mmol) and 9,10-dihydroanthracene (0.036 g, 0.20 mmol). The reaction mixture was analyzed by ^{1}H NMR spectroscopy after 45 minutes, which showed a 13% yield of anthracene using [^{n}Bu$_4$N]$^+$ as an internal standard.

UV-vis measurement of [^{n}Bu$_4$N]$_2$1 + O$_2$. Dry O_2 (1 atm) was added to a Teflon sealed, air-tight quartz cuvette containing a solution of [^{n}Bu$_4$N]$_2$**1** in 1:1 MeCN/THF (3.0 mL, 8.5 x 10^{-5} M) at −63°C. A measurement was taken every minute for 60 minutes from the point of O_2 addition. To record the spectrum of the completed reaction, the cuvette was then allowed to warm up to room temperature for 10 minutes, and then cooled back down to −63°C before the spectrum was recorded.

Preparation of [6]$^{2-}$ ESI-MS sample. Dry O_2 was added via a blunt syringe to a vial capped with a rubber septum containing a solution of $[Me_4N]_2$**1** in MeCN (1 mM). At room temperature, an aliquot from the reaction mixture was analyzed after approximately 30 s. Figure S2 shows the full spectrum and inset shows the zoomed up region for **[6]$^{2-}$.**

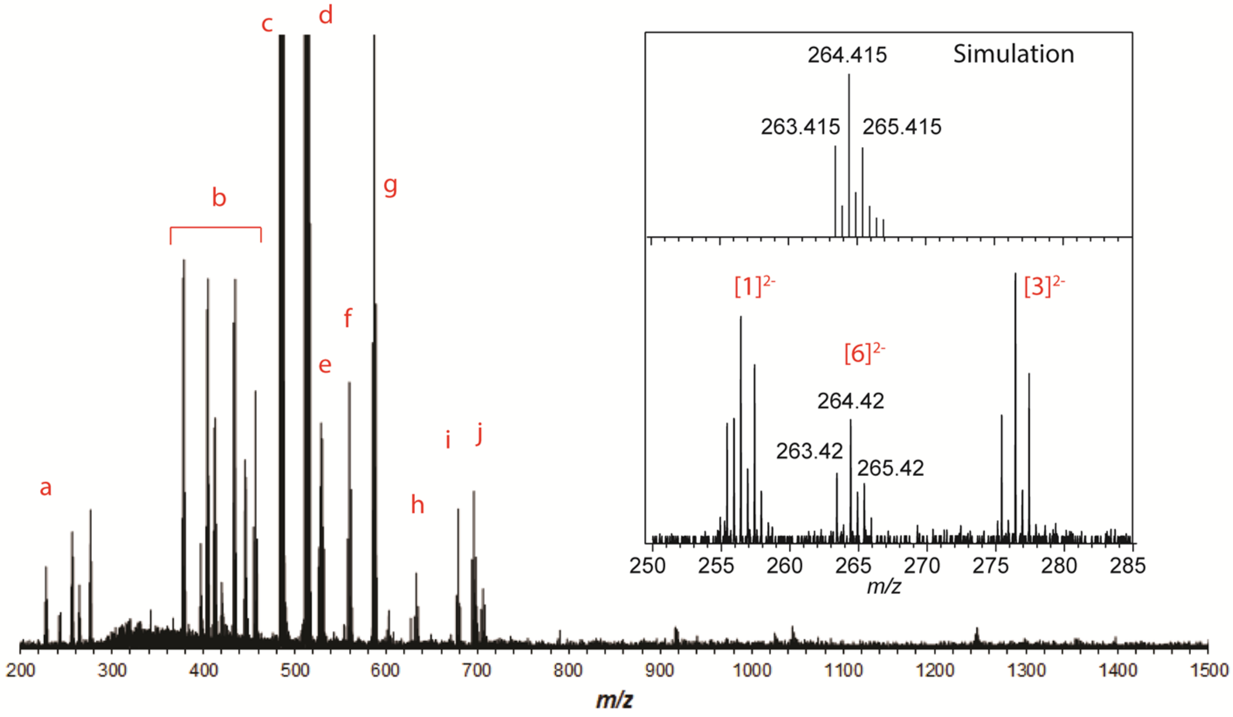

Figure S2. Negative-ion ESI-MS (full and close up of region-of-interest). Other ions identified are: (a) *227.96*, $[LH_2]^{2-}$, (b) *378.86*, $[LH_4(-Br)]^-$, (c) various monobrominated complexes, indicated by a doublet pattern (debromination in ESI), (d) *485.39,* $[(LH_2)_2Co^{II}]^{2-}$, (e) **[2]**$^-$, (f) *528.83*, $[LCoO]^-$, (g) *580.93*, $[(LCo^{II})(Me_4N)]^-$, (h) *632.93*, $[(LH)Co^{II}(formate)(Me_4N)]^-$, (i) *678.94*, $[(LH_2)Co^{II}(formate)_2(Me_4N)]^-$, (j) *696.04*, $[(LCo^{II})(H_2O)(OH)(Me_4N)_2]^-$. No peaks could be assigned to the species $[LCo^{III}OH]^{2-}$, $[(LCo^{III})_2O]^{4-}$, $[LCo^{III}O_2]^-$, $([LCo^{III})_2O_2]^{4-}$, or their adducts with either Na^+ or Me_4N^+. (L= **[^{Br}HBA-Et]$^{4-}$**)

**Note*: Relative abundance is not representative of relative concentrations in solution.

Preparation of [^{n}Bu$_4$N]$_2$1 EPR sample. A solution of [^{n}Bu$_4$N]$_2$**1** in ^{n}PrCN (5 mM) in a quartz EPR tube was prepared. The solution was cooled to 77 K. Figure S3 shows the simulated and experimental EPR spectrum.

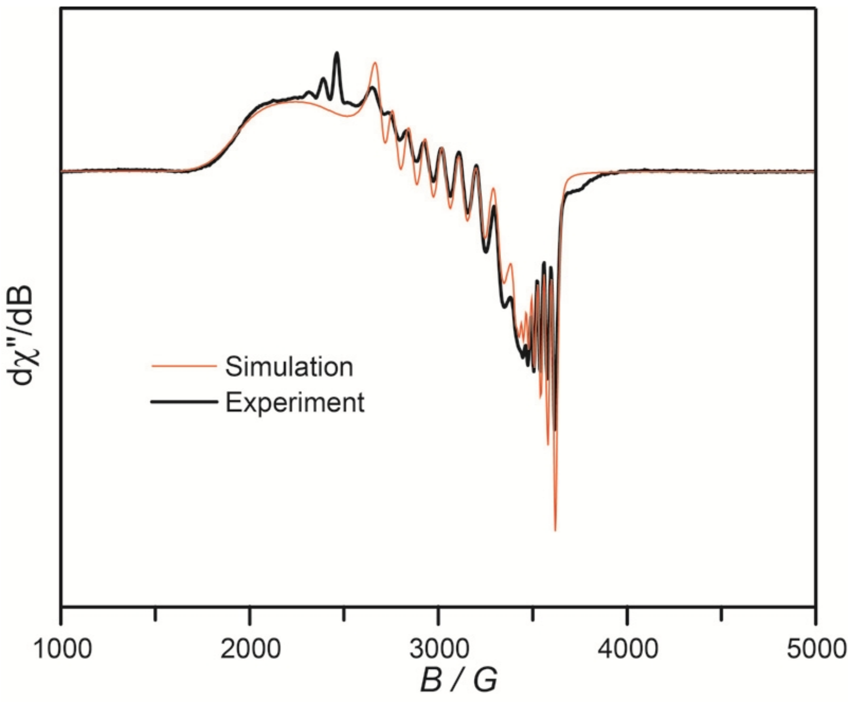

Figure S3. Simulated and experimental spectrum for **[1]$^{2-}$**.

Observation of [5]$^{2-}$ generated from O_2, by EPR. Dry O_2 was added to a quartz EPR tube capped with a rubber septum containing a solution of [^{n}Bu$_4$N]$_2$**1** in ^{n}PrCN (5 mM). The reaction mixture was maintained at room temperature for approximately 30 s and then was immediately cooled to 77 K. Figure S4 shows the simulated and experimental EPR spectrum.

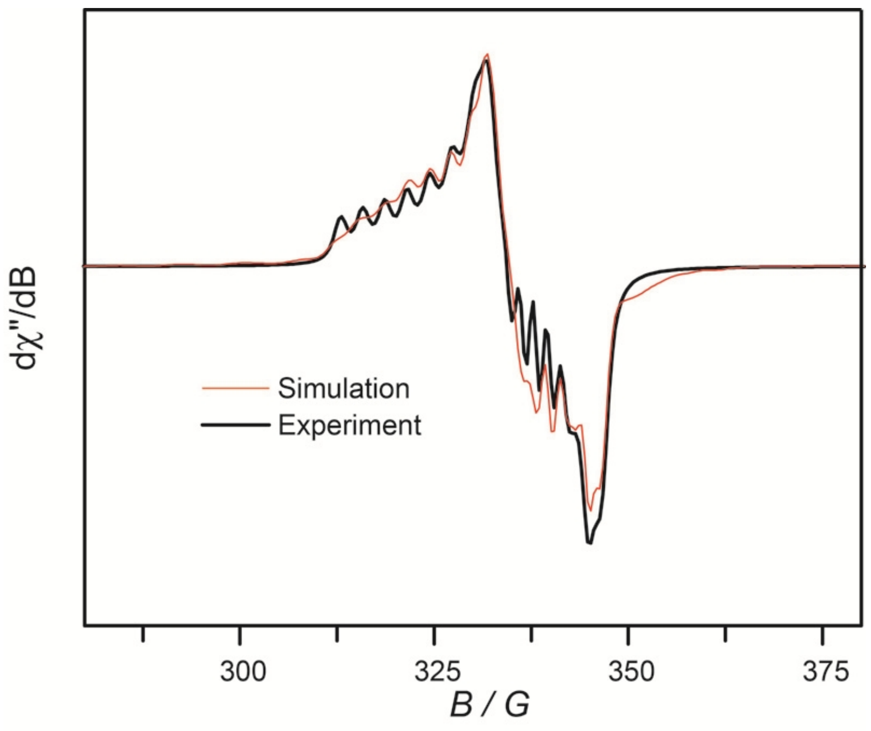

Figure S4. Simulated and experimental spectrum for **[5]**$^{2-}$.

Reversible formation of [5]$^{2-}$ observed by EPR. An EPR spectrum was acquired of a Teflon sealed J. Young tube containing a solution of [^{n}Bu$_4$N]$_2$**1** in MeCN (5 mM). The solution was degassed by one freeze-pump-thaw cycle and dry O_2 was added to the vessel and the reaction mixture was maintained at room temperature for approximately 60 s, and then it was rapidly cooled to 77 K and an EPR spectrum was acquired. The solution was then degassed by one freeze-pump-thaw cycle, cooled to 77 K, and an EPR spectrum was acquired. Figure S5 shows the overlay of the two spectra.

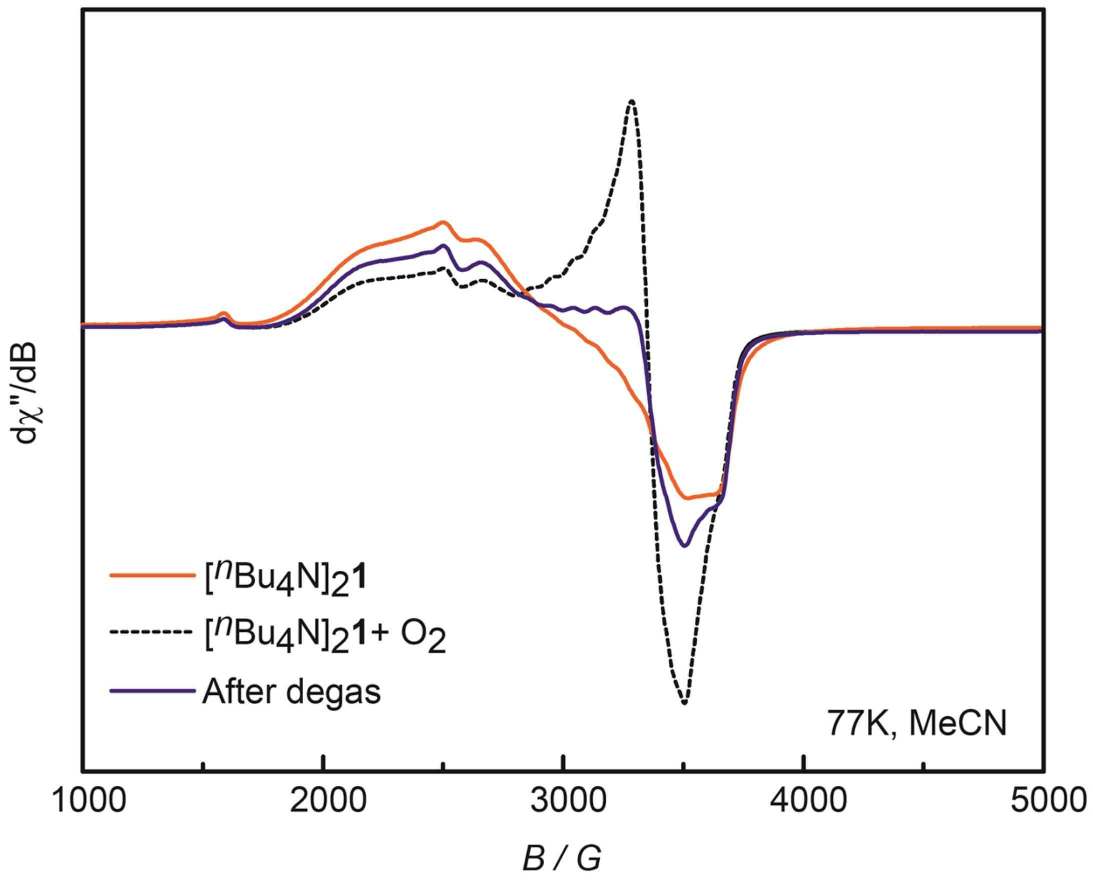

Figure S5. EPR spectra of the reaction mixture before O_2 addition, after O_2 addition, and after degassing.

Analysis of complete reaction mixture ([^{n}Bu$_4$N]$_2$1 + O_2) by EPR. Dry O_2 was added to a quartz EPR tube capped with a rubber septum containing a solution of [^{n}Bu$_4$N]$_2$**1** in ^{n}PrCN (5 mM). The reaction was allowed to occur at room temperature for approximately 10 min, and the reaction mixture was then rapidly cooled to 77 K.

Preparation of [^{n}Bu$_4$N]$_2$1 sample for cyclic voltammetry. A solution of **[1]$^{2-}$** (1.04 x 10^{-3} M, 10 mL) in 0.10 M tetra-*n*-butylammonium hexafluorophosphate acetonitrile solution was prepared in a N_2 filled glovebox at room temperature. Potentials were then referenced to ferrocene internal standard.

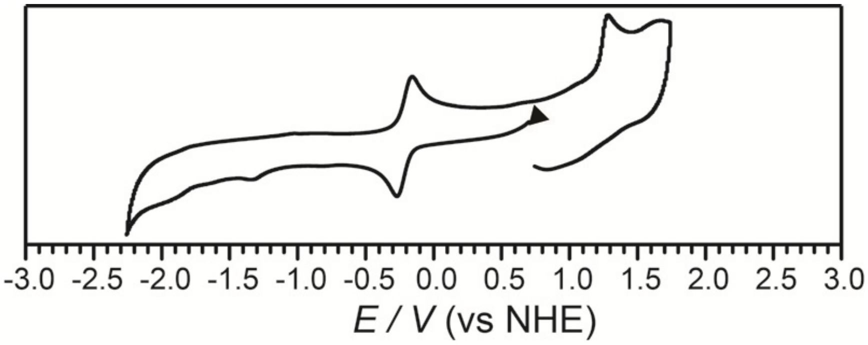

Figure S6. Cyclic voltammagram of [^{n}Bu$_4$N]$_2$**1** at 100 mv/s (without ferrocene).

3. X-ray Crystallography Details

X-ray diffraction data were collected using Bruker AXS three-circle diffractometers coupled to a CCD detector with either QUAZAR multilayer mirror- or graphite-monochromated Mo Kα radiation (λ = 0.71073 Å). The structures were solved by direct methods using SHELXS and refined against F^2 on all data by full-matrix least squares with SHELXL-97. All non-hydrogen atoms were refined anisotropically; hydrogen atoms were included into the model at their geometrically calculated positions and refined using a riding model.
